# Feedback to Prepare EMS Teams to Manage Infected Patients with COVID-19: A Case Series

**DOI:** 10.1017/S1049023X20000783

**Published:** 2020-06-08

**Authors:** Daniel Aiham Ghazali, Amina Ouersighni, Matthieu Gay, Virginie Audebault, Thomas Pavlovsky, Enrique Casalino

**Affiliations:** 1. Assistance Publique-Hôpitaux de Paris (AP-HP), Groupe Universitaire Paris Nord Val de Seine, EMS of Beaujon Academic Hospital, Paris, France; 2. Assistance Publique-Hôpitaux de Paris (AP-HP), Groupe Universitaire Paris Nord Val de Seine, Emergency Department of Bichat Academic Hospital, Paris, France; 3. Infection Antimicrobials Modelling Evolution (IAME) Research Center, UMR 1137 – INSERM, University of Paris Diderot, Paris, France

**Keywords:** COVID-19, emergency, EMS, personnel protection equipment, providers’ contamination

## Abstract

Coronavirus Disease 2019 (COVID-19), a new respiratory disease, is spreading globally. In France, Emergency Medical Service (EMS) teams are mobile medicalized resuscitation teams composed of emergency physician, nurse or anesthesiologist nurse, ambulance driver, and resident. Four types of clinical cases are presented here because they have led these EMS teams to change practices in their management of patients suspected of COVID-19 infection: cardiac arrest, hypoxia on an acute pneumonia, acute chronic obstructive pulmonary disease (COPD) exacerbation with respiratory and hemodynamic disorders, and upper function disorders in a patient in a long-term care facility. The last case raised the question of COVID-19 cases with atypical forms in elderly subjects. Providers were contaminated during the management of these patients. These cases highlighted the need to review the way these EMS teams are responding to the COVID-19 pandemic, in view of heightening potential for early identification of suspicious cases, and of reinforcing the application of staff protection equipment to limit risk of contamination.

## Introduction

Coronavirus Disease 2019 (COVID-19), a new respiratory disease, is spreading globally. COVID-19 was initially reported in late 2019 and was declared a pandemic on March 11, 2020. In France, at least 71,000 people are currently affected by the COVID-19 pandemic, and over 8,900 have already died.^1^ The Paris region is the most affected area in France, with at least 10,176 cases requiring hospitalization and 2,516 in intensive care units (ICUs). As front-line caregivers, Emergency Medical Services (EMS) teams are exposed to life-threatening events in patients requiring resuscitative management. It has been shown in Chinese series that nearly 30% of the patients hospitalized for a COVID-19 infection were providers who had been contaminated in their workplaces.^2^ French EMS teams are mobile medicalized resuscitation teams, generally composed of four members: emergency physician, nurse, ambulance driver, and resident or other trainee. The French prehospital system allows specialized resuscitations to begin before arrival at a hospital.

These clinical cases were chosen because they have led to changes in practices in the prehospital management of patients suspected of COVID-19 infection.

## Case Reports

### Case 1

One of the teams intervened for cardiac arrest in a 70-year-old patient with no known history. Initial monitored rhythm was asystolic. The patient was treated in accordance with the European Resuscitation Council (Niel, Belgium) 2015 Guidelines for resuscitation.^3^ Adrenaline 1mg was given as soon as venous access was achieved, and repeated every alternate cardiopulmonary resuscitation (CPR) cycle (ie, every four minutes). Intubation was performed after the second CPR cycle. Once this patient’s trachea had been intubated, the patient was ventilated at 10 breaths minute−1; associated with continuous chest compressions at a rate of 100-120 minute−1 without pausing during ventilation. No return of spontaneous circulation (ROSC) was observed and CPR was stopped after 30 minutes. During resuscitation, the team questioned the patient’s relatives: an infectious cause was suspected due to a fever of 39.5°C (103.1°F), a cough for three days, and dyspnea since the day before. The COVID-19 post-mortem reverse transcription polymerase chain reaction (RT-PCR) test, performed on suspicion of severe hypoxemia pneumonia, was positive. At Day 4 of this intervention, two team members (ambulance driver and nurse) were symptomatic and their RT-PCR test was positive. Between the intervention and the contamination, they were resting. During CPR, only the physician wore glasses and a surgical mask to intubate the patient.

### Case 2

Intervention in a 56-year-old patient with a history of chronic obstructive pulmonary disease (COPD) for acute respiratory failure. Pulse-oximetry oxygen saturation (SpO2) was 88% and then 93% SpO2 under oxygen at 5L/minute using oxygen mask. The patient presented skin mottling and blood pressure of 91/49mmHg corrected by 500mL 0.9% sodium chloride solution replacement. Noninvasive ventilation (Bilevel positive airway pressure; BiPAP) with 100% fraction of inspired oxygen (FiO2), then 60% FiO2 was set up with inspiratory positive airway pressure at 13cmH2O and expiratory positive airway pressure at 4cmH2O. The patient was placed in the ICU. The ICU team was contacted and the patient’s RT-PCR was positive. One team member was symptomatic at Day 5 of this procedure; COVID-19 RT-PCR was positive.

### Case 3

Intervention for acute respiratory failure with SpO2 70% in a 63-year-old patient with a notion of fever and dyspnea since the day before. On arrival, despite oxygen with a high concentration mask, SpO2 was 88%. It was decided to intubate the patient. The patient presented secretions requiring aspiration before intubation. During aspiration, there was a dissemination of secretions with projection observed. Intubation was difficult, performed in hypoxemic conditions despite pre-oxygenation of the patient and induction curarization using a short-acting neuromuscular blocking agent (Succinylcholine, 1mg/kg) associated with short-acting intravenous anesthetic for sedation (Etomidate 0.5mg/kg). The patient was transported to ICU. The patient’s COVID-19 RT-PCR was positive. One team member, the intubator, was symptomatic and had a positive COVID-19 RT-PCR. The patient is currently still in the ICU on mechanical ventilation.

### Case 4

Intervention in a long-term care facility for drowsiness in an 86-year-old patient. On arrival, the team noted a Glasgow Coma Score of 10 with additional confusion, according to the nurses. The patient had no fever or respiratory symptoms, but did suffer from abdominal pain and 85% SpO2. The patient was transferred to the emergency department (ED) on oxygen, with 93% SpO2 at 9L/minute using a high-concentration oxygen mask. On arrival, an abdominal computerized tomography (CT) scan was performed. The CT scan revealed bilateral and peripheral ground-glass opacities. COVID-19 RT-PCR was positive. The ED nurse who managed this patient was infected with COVID-19 five days later.

## Discussion

COVID-19 is an emerging, rapidly evolving situation throughout the world. This case series presents four types of clinical cases, which are very frequent during medicalized EMS interventions in France: cardiac arrest, hypoxia on acute pneumonia, acute COPD exacerbation with respiratory and hemodynamic disorders, and upper function disorders in a patient in a long-term care facility. These cases are illustrative of the difficulties faced by EMS teams in the initial phase of the COVID-19 epidemic in France. During these procedures, three members of the EMS teams and one ED nurse were contaminated with COVID-19. These cases demonstrate that compliance with the rules for the protection of staff working with patients is absolutely essential.

It has been noted that actions that are known to carry a risk of transmission of infectious respiratory agents, such as noninvasive ventilation and intubation,^4^ were the causes of the contamination of three members of the EMS teams. These cases represent a reminder of the importance of using personal protective equipment in all situations of cardiac arrest and respiratory distress, particularly in this epidemic context. It also matters to limit certain high-risk procedures to recognized indications.^4^ As of now, there is insufficient evidence to support or refute the use of any specific technique to maintain an airway and provide ventilation in adults with cardiopulmonary arrest.^3^ In today’s pandemic context, tracheal intubation should be avoided until ROSC is obtained. Similarly, the number of providers should be limited to avoid the risk of contamination.

The last case raises the question of COVID-19 cases with few or no respiratory symptoms, as well as COVID-19 cases with atypical forms. In this patient, there was no respiratory distress or initial polypnea, but rather confusion and abdominal pain. COVID-19 pneumonia was discovered incidentally on the CT scan. Recently, there have been reports on the diagnostic value of chest CT in COVID-19 patients.^5^ This patient indeed had abdominal pain, which has been described in the pneumonia of elderly subjects.^6^

Following these cases, the application of personnel protection equipment has been reinforced during patient management and transfer: systematic wearing of a surgical mask for all patient care, and systematic hand washing or application of hydroalcoholic solution. For patients with respiratory, hemodynamic, or neurological distress, and during a procedure with an increased risk of dissemination and transmission of respiratory infectious agents, providers must use glasses and disposable medical hair cover (scrub cap). They must wear nonsterile, disposable patient isolation gowns, which are appropriate for use by patients with suspected or confirmed COVID-19. They also must wear a respirator of at least class FFP2, and nonsterile gloves, in accordance with existing recommendations.^4^ In Europe, FFP2 masks must have a minimum of 94% filtration percentage and maximum 8% leakage to the inside. Finally, teams have been required to limit risky acts and to limit the number of providers involved in patient management. For intubation, if absolutely necessary, use of sedation and rapid sequence muscle relaxants is mandatory.^7^ If possible, a video laryngoscope and a closed suction system are recommended.

## Conclusion

This work indicates that review is needed in the way EMS teams respond to the COVID-19 pandemic in order to maintain a strong potential for early identification of suspicious cases and to ensure the safety of patients and staff of the EMS teams and the entire hospital. Any patient with signs of respiratory, hemodynamic, or neurological severity should be considered affected by the COVID-19 pandemic in the current epidemic context.
